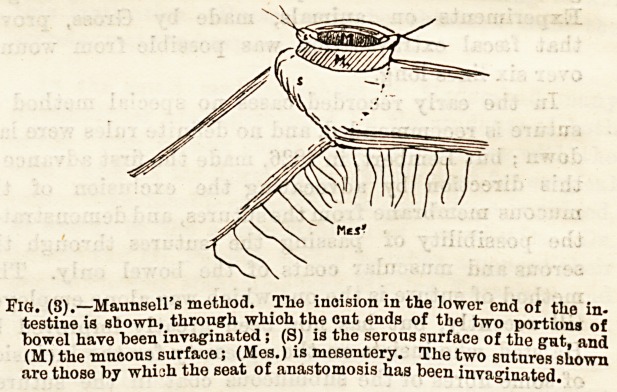# Intestinal Anastomosis

**Published:** 1895-05-11

**Authors:** Leonard A. Bidwell

**Affiliations:** Senior Assistant Surgeon to the West London Hospital


### Medical Progress and Hospital Clinics.

[*The Editor will be glad to receive offers of co-operation and contributions from members of the profession. All letters should be addressed to* The Editor, The Lodge, Porchester Square, London, W.]

### INTESTINAL ANASTOMOSIS.

By Leonard A. Bidwell, F.R.C.S., Senior Assistant Surgeon to the West London Hospital.

Now that surgeons have more confidence in opening the abdomen for the diagnosis of obscure abdominal pains, it is possible that a large number of cases of intestinal tumour may be safely removed. Physicians have been restrained from advising operation by the dread of the patient dying from peritonitis, but this untoward result, in my opinion, now very rarely occurs after a carefully performed laparotomy, in fact out of thirty consecutive cases of laparotomy for various causes I have not lost a single case from peritonitis.

All must admit that it is most necessary for any surgeon who undertakes a case of abdominal section, to be familiar with some method of intestinal anastomosis, since he can never be certain, even in the most simple case, that the intestine will not be injured. The choice of methods is now very great, but many of those recently introduced are only modifications or perfections of older ones. In criticising the various procedures at our disposal, we would divide them into methods of end to end, and of lateral approximation; we may further divide them into those effected by mechanical appliances and those done solely with sutures. Speaking generally, mechanical appliances are not so trustworthy as sutures, and of course the apparatus may not be at hand when it is wanted.

Although it is only of recent years that intestinal anastomosis has been performed with any certainty of success, a recovery, after resection of intestine, was recorded in 1730. B. Bell also, in 1783, strongly urged the necessity for intestinal suture in all wounds of gut, even when under a quarter of an inch in length. Experiments on animals, made by Gross, proved that fæcal extravasation was possible from wounds over six lines long.

In the early recorded cases no special method of suture is recommended, and no definite rules were laid down; but Lembert, in 1826, made the first advance in this direction by advocating the exclusion of the mucous membrane from the sutures, and demonstrated the possibility of passing the sutures through the serous and muscular coats of the bowel only. This method of suture is the one which was alone employed till recently, but has now been greatly improved by Halsted, who insists on the necessity for the inclusion of some fibres of the submucous coat in the sutures, since the submucous coat is the most resistant of all the coats, and sutures passed through the serous and muscular alone readily cut out.

Recently the principle of invagination has been revived by the late Dr. Maunsell, who recommended an excellent method which will be described later. The principle, however, is an old one, and was advanced by Jobert, who recommended a special stitch which produced invagination of the edges of the bowel, the stitches being passed from outside. His results, however, do not appear to have been satisfactory.

With regard to mechanical appliances, the following are those now in use: (1) Senn's decalcified bone plates, or their modifications; (2) Mayo Robson's bone bobbin; (3) Paul's bone tube for invagination; (4) Murphy's button.

The chief advantages of any of these methods are the rapidity of their execution and the small number of stitches (if any) required. All these methods, however, appear to be followed by contraction of the opening, and in not a few cases there has been leakage from the joint. Murphy's button is theoretically very simple and efficient, and its application takes only a few minutes. Although almost invariable success was claimed for its use in the hands of its inventor, in the hands of other surgeons fæcal extravasation has followed in several cases. That a patient's life should depend on the good action of a spring, as it does when this button is used, seems rather a serious matter. Besides which if a surgeon was accustomed to the use of Murphy's button, and was unfamiliar with any other method of anastomosis, it would be very awkward, both for him and for the patient, if he were suddenly called upon to do an intestinal anastomosis when he had no button at hand. In all operations on the intestines experience in a particular method of operating is most important, and greatly influences the result; in fact, each surgeon is most successful in using the method which he knows best.

There are three chief points to be considered in affecting an anastomosis, or in suturing a wound of intestine.

(1) That adequately broad and sufficiently wide surfaces of healthy intestine should be in contact.

(2) That though it is advisable to exclude the mucous membrane from the stitches, the fibres of the submucous coat must always be included.

(3) That the operation should be performed as rapidly as possible.

I have no hesitation in recommending the methods of Halsted and of Maunsell as the simplest and best for all wounds and anastomoses of intestine. I will, therefore, give a brief description of their mode of performance.

Halsted's method effects a lateral anastomosis of intestine, and is therefore best suited for cases of gastro-jejunostomy or for short circuiting a loop of intestine, where a more radical operation cannot be attempted; it is also admirably adapted for the closing of all wounds of intestine. I believe that its performance is free from risk of fæcal extravasation when properly carried out.

Maunsell's method, on the other hand, is best adapted for end to end anastomoses, and effects a very good joint; its only fault being the fact that the sutures pass through all the coats of the gut, and so peritoneal infection from the mucous membrane along the stitch-tracts is possible.

Halsted gives the following directions for performing anastomosis, and the woodcuts after Halsted will help to make the description clear :—

" Six square or quilt sutures are inserted in a straight row near the mesenteric borders of the knuckles of intestine and are tied (as in Fig. 1). At each end of this posterior row of sutures, and nearer the convex border of the intestine, two lateral square stitches are employed (as in Fig. 1), and are tied. A little beyond the convex border of the gut, the eight or nine square stitches which constitute the anterior row, and complete the

FIG. (1).—The two coils of intestine side by side; the six posterior sutures have been passed and tied; the two at the top and bottom have been passed but not tied. (After Halsted).

FIG. (2).—All the sutures have been passed, the posterior and end sutures have been tied; the eight anterior sutures have been drawn aside in order to make the openings into the gut; these are shown; the operation is completed by rapidly tying the anterior sutures. (After Halsted.)

oval, are applied but not tied. They are then drawn aside to make room for the knife or scissors with which the two approximated intestines are then opened (as shown in Fig. 2). The sutures of the anterior row are tied under a constant and gentle irrigation with tepid salt solution, 6 in 1,000, which is poured from the flask in which it has been sterilised."

Each stitch should include a bit of the submucosa; since a thread of this coat is much stronger than a shred of the entire thickness of the serosa and muscularis. It is not difficult to familiarise oneself with the resistance furnished by the submucosa; the needle should be introduced by pressing the blunt end with the pulp of one of the fingers, when it will be found easy to pick up a bit of this coat with each stitch. The best needle for this purpose is the so-called straw needle of the size of No. 8.

The second method of anastomosis, which I recommend, is that devised by Maunsell. Its method of performance is as follows:—

An incision is made in the convex border of the gut just below the part to be sutured, and two sutures are placed in the mesenteric and convex surfaces of the cut ends; these sutures are tied and their ends dragged through the incision made in the convex border of the intestine. By pulling on these the lower part of the bowel is invaginated on the upper, and the cut ends are made to present through the incision in the gut (as shown in Fig. 3). The cut ends are then united by interrupted sutures which are passed through all the coats of the gut, and are tied tightly; they are placed about one-eighth of an inch apart and the ends are cut off short. The invagination is then reduced; the opening in the gut below the joint is closed with three or four Halsted's stitches.

The following has been my experience of these two methods of suture :—

Case 1.—Man, aged 67 years. Gastro-jejunostomy, for cancer of pylorus, by Halsted's method for lateral anastomosis. Recovery from the operation and relief of symptoms, but death six weeks later from exhaustion.

Case 2.—Female, aged 30 years. Gastro-jejunostomy, for pyloric cancer, by Halsted's method. Patient was in a desperate condition, and died on the tenth day from broncho-pneumonia. Anastomosis acted well, and patient took plenty of nourishment. There was no sign of any peritonitis, and the opening was soundly healed.

Case 3.—Female, aged 27 years. Rupture of small intestine while separating adhesions in operation for extra-uterine fœtation. Five inches of damaged intestine resected, and ends of gut joined by Maunsell's method. Complete recovery.

Case 4.—Man, aged 45 years. Simple stricture of pylorus, with symptoms for twenty years. Incision of stomach and digital dilatation of pylorus. Wound in stomach closed with six Halsted's sutures passing through the sub-mucous coat. Recovery without a single bad symptom. Food given the day after operation.

During the last few years a considerable number of successful cases of intestinal suture have been recorded. In some Senn's plates, and in others Lembert's suture have beenemployed, and a few, only, have been done by the methods which I have advocated. It is impossible to calculate the number of unsuccessful cases which have been done and have not been recorded, for we all know that surgeons are more ready to publish successes than failures.

There are certain conditions in which intestinal suture is not likely to be succcessful. These are—(1) In advanced cases of cancer; (2) in cases of paralytic dilatation of intestine from acute obstruction; and (3)

FIG. (3).—Maunsell's method. The incision in the lower end of the intestine is shown, through which the cut ends of the two portions of bowel have been invaginated; (S) is the serous surface of the gut, and (M) the mucous surface; (Mes.) is mesentery. The two sutures shown are those by which the seat of anastomosis has been invaginated.

when the sutures pass through bruised or otherwise damaged tissues. In fact, we must have healthy tissues to work upon.

To secure success in these cases it is necessary to mitigate the shock attending the operation by every means in our power. This can best be done by operating upon a table artificially heated by hot water or steam. One need hardly refer to the necessity, when possible, of clearing out the bowels for two days before the operation, and we might mention the suggestion which has been made of attempting to aseptisise the intestinal contents by the administration of salol or beta-naphthol for several days before the operation. This I have done in several of my cases.

With regard to after-treatment, early feeding is a most important indication, even after an operation on the stomach, in which case food should be given by the mouth after eighteen hours. Rectal feeding may be commenced a few hours before the operation, and must be continued until the patient is taking a good quantity by the mouth. The routine use of opium in abdominal surgery cannot be too strongly condemned; it should only be given in severe pain.

In my cases of gastro-enterostomy I have given hot water only for the first twelve hours, and have then commenced barley water in one ounce doses for the next twelve hours. After this drinks of peptonised milk and beef tea are allowed, together with a little Brand's essence or Valentine's meat juice; seventy-two hours after the operation the nutrient enemata are discontinued, and the quantity of fluids by the mouth are not limited. Custard pudding is given on the fourth day, and fish on the fifth. The only article of diet which I withhold is bread, which I do not allow for ten days. It has often been noticed in cases of typhoid fever that a relapse has occurred after bread has been given, but not after meat, &c. It has occurred to me that some of the cases which are supposed to have been lost by too early feeding may have been affected by the bread given. At any rate, in an intestinal suture case it is not worth while to run any risk, and so I always withhold bread.

Personally I have never seen any bad results from the practice of early and free feeding, but I can recollect many cases which have died from exhaustion, and might, in my opinion, have been saved by a more liberal diet.

## Figures and Tables

**Fig. (1) f1:**
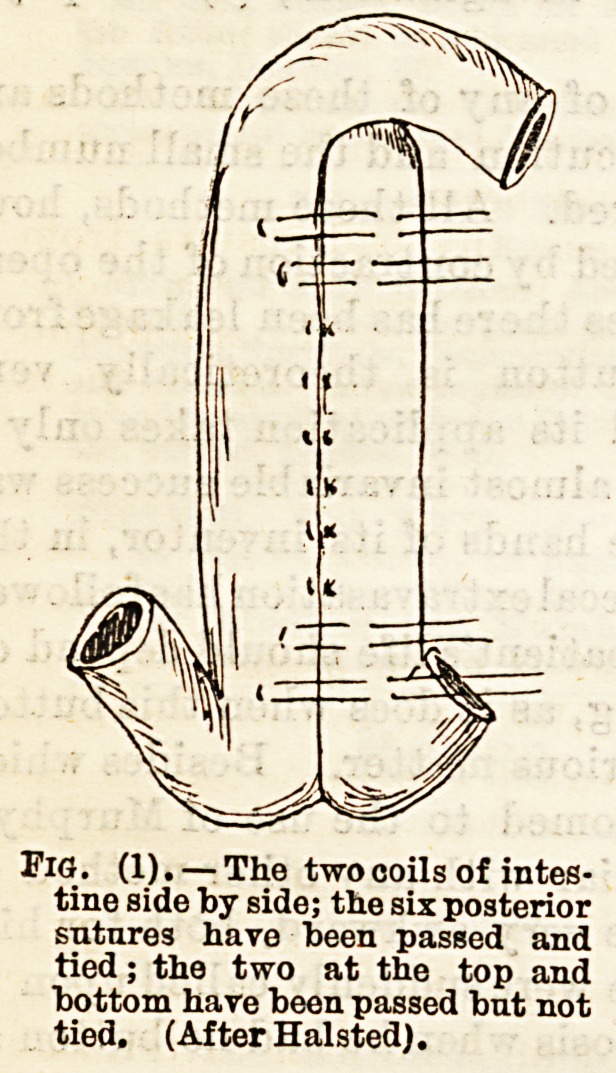


**Fig. (2) f2:**
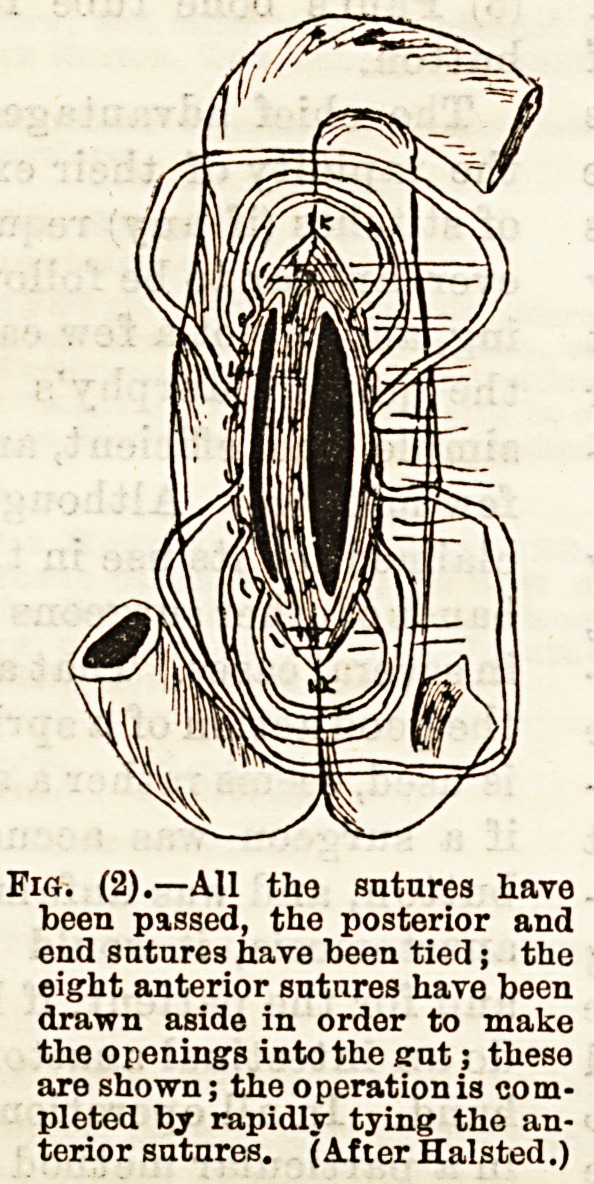


**Fig. (3) f3:**